# Cytogenetic Analysis of Satellitome of Madagascar Leaf-Tailed Geckos

**DOI:** 10.3390/genes15040429

**Published:** 2024-03-28

**Authors:** Alona Yurchenko, Tomáš Pšenička, Pablo Mora, Juan Alberto Marchal Ortega, Antonio Sánchez Baca, Michail Rovatsos

**Affiliations:** 1 Department of Ecology, Faculty of Science, Charles University, 128 44 Prague, Czech Republic; alona.yurchenko@natur.cuni.cz (A.Y.); tomas.charvat@natur.cuni.cz (T.P.); 2 Department of Experimental Biology, Faculty of Experimental Sciences, University of Jaén, Campus Las Lagunillas s/n, E-23071 Jaen, Spain; pmora@ujaen.es (P.M.); jamaor@ujaen.es (J.A.M.O.); abaca@ujaen.es (A.S.B.)

**Keywords:** evolution, FISH, Gekkonidae, karyotype, RepeatExplorer, satellite DNA

## Abstract

Satellite DNA (satDNA) consists of sequences of DNA that form tandem repetitions across the genome, and it is notorious for its diversity and fast evolutionary rate. Despite its importance, satDNA has been only sporadically studied in reptile lineages. Here, we sequenced genomic DNA and PCR-amplified microdissected W chromosomes on the Illumina platform in order to characterize the monomers of satDNA from the Henkel’s leaf-tailed gecko *U. henkeli* and to compare their topology by in situ hybridization in the karyotypes of the closely related Günther’s flat-tail gecko *U. guentheri* and gold dust day gecko *P. laticauda*. We identified seventeen different satDNAs; twelve of them seem to accumulate in centromeres, telomeres and/or the W chromosome. Notably, centromeric and telomeric regions seem to share similar types of satDNAs, and we found two that seem to accumulate at both edges of all chromosomes in all three species. We speculate that the long-term stability of all-acrocentric karyotypes in geckos might be explained from the presence of specific satDNAs at the centromeric regions that are strong meiotic drivers, a hypothesis that should be further tested.

## 1. Introduction

Satellite DNA (satDNA) consists of sequences of DNA that form tandem repetitions across the genome. Arrays of satDNA are abundant in the genome of all eukaryotic organisms and, according to the size of the monomer, are often categorized as microsatellites, minisatellites, satellites and macrosatellites [1,2]. satDNA is typically abundant in heterochromatic regions but can also be detected in euchromatic regions of the genome [3,4,5,6]. Extensive arrays of satDNA are typically located in centromeres, telomeres, supernumerary chromosomes and sex chromosomes [1,7,8,9]. The satDNA arrays are among the fastest evolving regions; multiple types of satDNA might coexist in the genome, and they tend to vary significantly in nucleotide sequence, copy number or topology even among homologous genomic regions of closely related species or even conspecific individuals [3,10,11,12]. Several mechanisms have been proposed to explain this variability, including strand slippage during DNA replication, unequal exchange, insertion or synthesis by DNA repair mechanisms, gene conversion, rolling circle replication and transposition together with transposable elements [1,3,11,13,14,15,16]. Certain types of satDNAs are often associated with fragile sites and evolutionary breakpoints that cause chromosomal rearrangements (e.g., fusions, fissions, inversions) and may play an important role in karyotype morphology and evolution [17,18,19]. For example, certain centromeric satellites, such as the minor satDNA in mouse, seem to favor Robertsonian fusions [20,21], while interstitial telomeric repeats are often considered as hotspots of chromosome breakage [22,23].

Satellite DNA arrays do not typically code for proteins; therefore, early studies considered them as useless genomic elements and assigned them as “junk” or “parasitic” DNA [24,25]. This view has gradually changed after accumulating evidence that satDNA seems to play an important role in the function and evolution of the genome. Few such prominent examples are the telomeric repeats that protect the chromosome edges from degradation [22,23] or the centromeric repeats that interact with DNA-binding proteins for the formation of the kinetochore and chromosome segregation during mitosis and meiosis [26]. In addition, recent studies provided evidence that certain satDNAs are crucial for the heterochromatin formation [27,28,29] or can actively regulate the expression of protein coding genes and even transcribe into non-coding RNA with various functions [4,30,31].

Recent studies revealed that the genome of squamate reptiles, the species-rich group with more than 10,000 species [32], has extensive accumulation of satDNAs [33,34]. Different types of satDNAs have been identified mainly in lacertids [35,36,37,38,39,40,41,42,43,44,45,46], skinks [44,47], snakes [48,49,50,51] and varanids [52,53], while interstitial telomeric-like repeats have been identified in almost half of the studied species of squamates [54,55,56,57,58,59]. Notably, despite that the sex-determination system remained stable in the long-term in snakes [60], lacertids [61,62] and varanids [63], their differentiated W chromosomes tend to accumulate satDNAs with extreme variation in topology and copy numbers among even closely related species [56,64,65,66].

Beyond lacertids, skinks, snakes and varanids, the repetitive content of sex chromosomes in reptiles was previously examined mainly by the topology of microsatellite repeats of a 1–5 nucleotide motif [64,67,68,69]. In the current study, we focused on geckos, a species rich group of which we have limited knowledge on satDNA content. We sequenced, on the Illumina platform, genomic DNA and amplified microdissected W chromosome DNA in order to identify satDNAs from the Henkel’s leaf-tailed gecko *U. henkeli* and to explore the topology of these satDNAs by in situ hybridization in the karyotypes of the closely related Günther’s flat-tailed gecko *U. guentheri* and gold dust day gecko *P. laticauda*. The leaf-tailed geckos of the genus *Uroplatus* and day geckos of the genus *Phelsuma* are small- to medium-sized lizards from the family Gekkonidae, both endemic to Madagascar. The leaf-tailed geckos seem to share a homologous ZZ/ZW sex-determination system, which emerged approximately 40 million years ago [70]. The W chromosome of *Uroplatus* geckos is large-sized, degenerated and heterochromatic with prominent heterochromatic blocks at the centromere, telomere and interstitial position [70], which indicates that it might be enriched in repetitive elements. On the contrary, all the studied geckos of the genus *Phelsuma* have temperature-dependent sex determination and, therefore, should lack sex chromosomes and have limited accumulation of repetitive elements.

## 2. Materials and Methods

### 2.1. Studied Material

Blood samples were collected from three species of geckos of the family Gekkonidae: a female of *U. henkeli*, both sexes of *U. guentheri* and a female of *P. laticauda*. The species and sex identification were based on external morphology. All studied animals were captive bred. The blood samples were collected from the brachial vein of the front legs and were used for DNA isolation and preparation of chromosome suspensions.

### 2.2. Chromosome Suspensions

Chromosome suspensions were obtained from whole-blood cell cultures [71]. The medium used for the cultures consisted of 90 mL D-MEM cell culture medium (without glucose, L-glutamine and sodium pyruvate; GIBCO, Carlsbad, CA, USA), 10 mL of fetal bovine serum (GIBCO, Carlsbad, CA, USA), 3 mL of phytohemagglutinin M (GIBCO, Carlsbad, CA, USA), 1 mL of penicillin/streptomycin solution (10,000 units/mL; GIBCO, Carlsbad, CA, USA), 1 mL L-glutamine solution (200 mM; Sigma-Aldrich, St. Louis, MO, USA) and 1 mL lipopolysaccharide solution (10 mg/mL; Sigma-Aldrich, St. Louis, MO, USA). Approximately 100–200 μL of blood was added to 5 mL of fresh medium and incubated for one week at 30 °C. After the incubation period, the mitotic cycle was arrested in metaphase by adding 35 μL of colcemid solution (10 μg/mL; Roche, Basel, Switzerland) to each sample. Following an incubation period of 3.5 h at 30 °C, the samples were centrifuged at 1200 rpm for 10 min at room temperature and incubated with 0.075 M of prewarmed KCl for 10 min at 37 °C. The blood cells were centrifuged again at 1200 rpm for 10 min at 4 °C, resuspended with 5 mL of fixative (3:1 methanol:acetic acid) and incubated for 20 min at 4 °C. The last step was repeated two additional times for better fixation, and the chromosome suspensions were stored at −20 °C until further use.

### 2.3. qPCR Validation of Sex Chromosome Homology

Total DNA of all individuals was isolated using the DNeasy Blood and Tissue Kit (Qiagen, Hilden, Germany). Previously published primer pairs for six Z-specific genes (*bsg*, *cdc73*, *dot1l*, *eps15*, *hectd3*, *lrrc41*) of *U. henkeli* [70] were used to verify the homology of sex chromosomes with *U. guentheri*. The method is based on the fact that ZZ males should have twice as many copies of Z-specific genes than ZW females, and this difference in gene copies between sexes can be measured by quantitative polymerase chain reaction (qPCR). If the primers show Z-specific values in both species, we can conclude that the Z chromosomes of both species are homologous.

### 2.4. Next-Generation Sequencing

Total DNA from a male and a female *U. henkeli* and two pools of amplified DNA of microdissected W chromosomes from this species were sequenced on the Illumina HiSeq2500 platform with 150 bp paired-end option (DNA-seq) and 350 bp library size at Novogene (Cambridge, UK). The raw Illumina reads are now available in the NCBI database (BioProject PRJNA1027145). The Illumina reads were trimmed for adapters and low-quality bases using Trimmomatic v0.39 [72]. The quality of the Illumina reads was checked in Fastqc v0.12 (www.bioinformatics.babraham.ac.uk/projects/fastqc/, accessed on 21 September 2022).

### 2.5. Repetitive Elements Analysis

The first stage of the analysis was to select random 12 million paired Illumina reads from each sample (i.e., sequences from total DNA from a male and a female *U. henkeli* and two pools of microdissected W chromosomes) using the *seqtk* package (https://github.com/lh3/seqtk, accessed on 21 September 2022). The Illumina reads were analyzed for repetitive motifs in RepeatExplorer with the default recommended options [73,74]. The putative satellite sequences were manually checked, compared and aligned in Geneious Prime v2022.1 (https://www.geneious.com/, accessed on 21 September 2022). The putative satellites were compared with the sequences in the nucleotide database of NCBI using *blast* [75]. In order to examine if these putative satellite sequences are in tandem, we created 5-mer sequences from each, and we mapped the Illumina reads in Geneious Prime v2022.1 (mapping parameters are provided in Table S1). If the repetitive motif is indeed in tandem, then the Illumina reads should map evenly across the mer connection points. RepeatMasker v4.1.16 [76] was used to estimate the abundance and divergence of satDNA monomers in the genome of *U. henkeli*.

### 2.6. Chromosome Staining and Karyotype Reconstruction

Chromosome suspensions were dropped onto glass slides and incubated at 60 °C for either 1 h or overnight prior to all cytogenetic experiments. The chromosome spreads were stained with 5% Giemsa solution (Penta Chemicals, Prague, Czech Republic) for 15 min to prepare the karyograms. C-banding stain was used to detect heterochromatin distribution and the W chromosome in *Uroplatus* following the protocol of Sumner [77]. Briefly, the chromosome spreads were first treated with 0.2 N HCl for 15 min at room temperature, then treated with prewarmed 5% Ba(OH)_2_ for 10 min at 45 °C and finally treated with 2 × SSC for 1 h at 60 °C. Subsequently, the chromosome spreads were washed with distilled water, air-dried and stained with Fluoroshield mounting medium with DAPI stain (Sigma-Aldrich, St. Louis, MO, USA).

### 2.7. Chromosome Microdissection

W chromosomes were dissected by sterile glass needles from C-banded chromosome spreads of *U. henkeli* using a Zeiss Axiovert S200 inverted microscope (Oberkochen, Germany) equipped with an Eppendorf TransferMan NK2 mechanical micromanipulator (Hamburg, Germany). The W chromosome of *U. henkeli* is acrocentric and similar in size to the three largest pairs of autosomes [70], but it is heterochromatic and, therefore, could be identified under the microscope after C-banding stain. In total, 20 W chromosomes were dissected twice independently, and the chromosomal material was amplified with the GenomePlex^®^ single cell whole genome amplification (WGA4) kit (Sigma-Aldrich, St. Louis, MO, USA). Part of the amplified microdissected material was labeled with Tetramethyl-Rhodamine-5-dUTP (Roche, Basel, Switzerland) by random primed DNA labeling Kit (Roche, Basel, Switzerland) to prepare a probe for in situ hybridization (i.e., chromosome painting). The specificity and strength of in situ hybridization were used as validation that the W chromosome was successfully microdissected and amplified (Figure S1). The rest of the amplified microdissected material was used for sequencing on the Illumina platform.

### 2.8. Fluorescence In Situ Hybridization

We applied fluorescence in situ hybridization to detect the topology of the putative satellites in the karyotype. We designed probes for all putative satellites that were identified from RepeatExplorer (Table S2). The probes were designed to detect the sequence of the first 32 bp from each putative satellite and were commercially synthesized and terminally labeled with biotin by Macrogen (Seoul, Republic of Korea).

The FISH experiments followed a standard protocol mentioned in [71]. The slides were subsequently treated with RNAse A (100 μg/mL) for 1 h at 37 °C, treated with 0.01% pepsin in PBS for 10 min at 37 °C and then post-fixed with 1% formaldehyde solution in PBS for 10 min and dehydrated in a 70–85–95% ethanol series for 5 min each at room temperature. After the chromosome spreads dried, they were denatured in 70% formamide for 3 min at 73 °C and dehydrated once more in a 70–85–95% ethanol series for 5 min each at room temperature. In parallel, for each slide and putative satellite motif, 0.3 μL of the commercial synthesized probe (stock solution of 100 pmol/µL) was mixed with 10 μL of hybridization buffer (50% formamide in 2 × SSC), denatured for 6 min at 73 °C and kept on ice for at least 10 min. The probes were applied to the chromosome spreads and incubated at 37 °C for overnight hybridization. During the second day, the chromosome spreads were washed in 0.4 × SSC/0.3% Nonidet P40 for 2 min at 55 °C and in 2 × SSC/0.1% Nonidet P40 for 30 sec at room temperature in order to remove the excess of probe and limit non-specific hybridization. The chromosome spreads were incubated in 4 × SSC/5% blocking reagent (Roche Diagnostics, Basel, Switzerland) for 45 min at 37 °C and then in 4 × SSC/5% blocking reagent containing avidin–FITC (Vector Laboratories, Burlingame, CA, USA) for 30 min at 37 °C. The fluorescence signal was enhanced twice using an avidin–FITC/biotinylated anti-avidin system (Vector Laboratories, Burlingame, CA, USA). The excess of antibodies was washed twice with 4 × SSC/0.1%Tween20 (Sigma-Aldrich, St. Louis, MO, USA) and once with PBS for 5 min each, and the chromosome spreads were dehydrated in a 70–85–95% ethanol series, dried and stained with Fluoroshield mounting medium containing DAPI (Vector Laboratories, Burlingame, CA, USA).

After capturing images of selected metaphases, the chromosome spreads were sequentially washed twice in 4×SSC/0.1%Tween20, twice in 2 × SSC and once in PBS for 5 min, dehydrated in an ethanol series for 5 min each and stained with C-banding in order to verify if the putative satellite motifs are located at the W chromosomes of *U. henkeli*.

### 2.9. Microscopy and Image Analyses

Images of selected metaphases were captured with an Olympus BX53 digital upright fluorescence microscope equipped with a 21-megapixel high-resolution Olympus DP74 color camera (Olympus, Tokyo, Japan). The images from the in situ hybridization experiments were combined and edited using Olympus DP manager v2.1.1.163 software. Karyograms were created using Adobe Photoshop CS6.

## 3. Results

In total, we identified 24 putative satDNA monomers by RepeatExplorer from genomic DNA and microdissected W chromosome sequences (Table S2). Comparisons with blast showed local similarity (i.e., top 100 hits) with sequences from reptiles (fifteen sequences), human (five sequences), and olive tree (one sequence); although, similarity was not revealed for three satDNA monomers (Table S2). Notably, the putative satellite motifs with similarity to human and olive tree were identified in the microdissected W chromosome samples but not in the total DNA samples. This was confirmed not only by the results of RepeatExplorer but also by mapping the Illumina reads from each sequenced DNA sample to the sequences of the monomer and the pentamer putative satellite motifs. The amount of amplified DNA from 20 microdissected W chromosomes is extremely reduced and has lesser quality/integrity as a consequence of the banding-related chemical treatments compared with DNA extracted from fresh blood. Therefore, we speculate that sequences with blast similarity to non-reptilian taxa might originate from random environmental DNA molecules (human contamination, olive tree pollen), which were unintentionally collected during microdissection and amplified by the WGA4 kit.

In order to check if these motifs are in tandem in the genome, we created 5-mer sequences for each putative satellite monomer and mapped the Illumina reads from the female total DNA sample. The Illumina reads appear to map evenly across the connection points in 17 motifs, indicating that they are probably located in tandem arrays in the genome, and therefore, they are verified satellite DNA motifs (including the fifteen with similarity with sequences from reptiles and two that did not show similarity with other sequences by blast comparison). The remaining seven motifs were excluded from further analysis as either contaminations of the microdissected material or not true satellites (Table S2).

The satDNA families were named according to Ruiz-Ruano et al. [78], including the species name abbreviation, a number in decreasing order of abundance and the length of the repeat sequence monomer starting from UhenSat01-129 (the most abundant) to UhenSat17-31 (the least abundant) (Table 1 and Table S2). The satellitome of *U. henkeli* is composed of at least 17 satDNA families, corresponding to 2.534% of the genome (Table 1 and Table S2). The proportion of satellitome in the genome shows a great variation among animal species from less than 1% to more the 50% of the genome (review in [79]), and therefore, the satellitome of *U. henkeli* is located in the lower part of this variability. The first two families comprise 0.9% of the genome; the following six families comprise 1.404% of the genome, while the remaining nine satDNAs represent only 0.23% (Table 1 and Table S2).

The mean A + T of the satDNA content is 51.79% with variation between 41.9% at UhenSat17-31 and 70.5% at UhenSat11-61 (Table 1 and Table S2), lower than the overall genome A + T content (54.8%) of *U. henkeli*, which is similar to the A + T content observed in the genomes of other gekkonid species, such as *Gekko japonicus* (54.5%; [80]) or *Paroedura picta* (55.5%; [81]).

The mean of the monomer length is 550.53, although high variation existed between the two largest ones with 4136 bp (UhenSat6-4136) and 3689 bp (UhenSat7-3689) and the smallest with 29 bp (UhenSat10-29). The mean of the monomer length, excluding the two largest ones, is 102.27, being the monomer length of the most abundant satellite DNA of 129 bp (UhenSat1-129).

**Figure 1:**
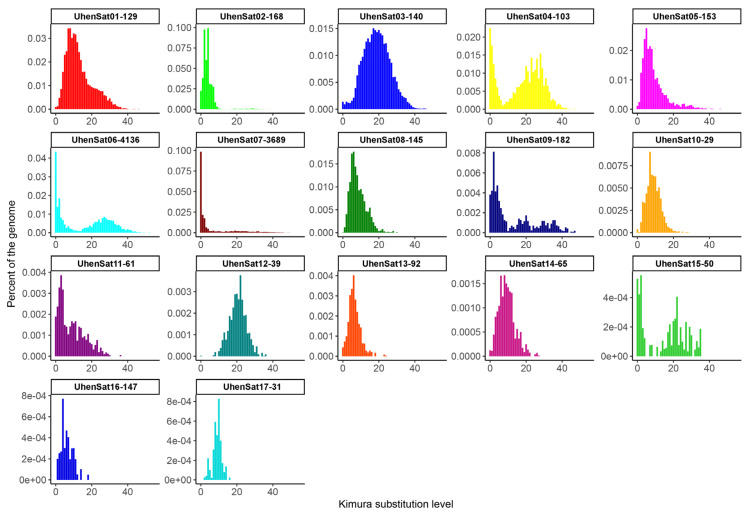
The satDNA landscape of *U. henkeli* showing the comparative abundance of the 17 satDNA families as the proportion of the female genome for each satDNA family versus the Kimura substitution divergence (both as percentages).

**Figure 2:**
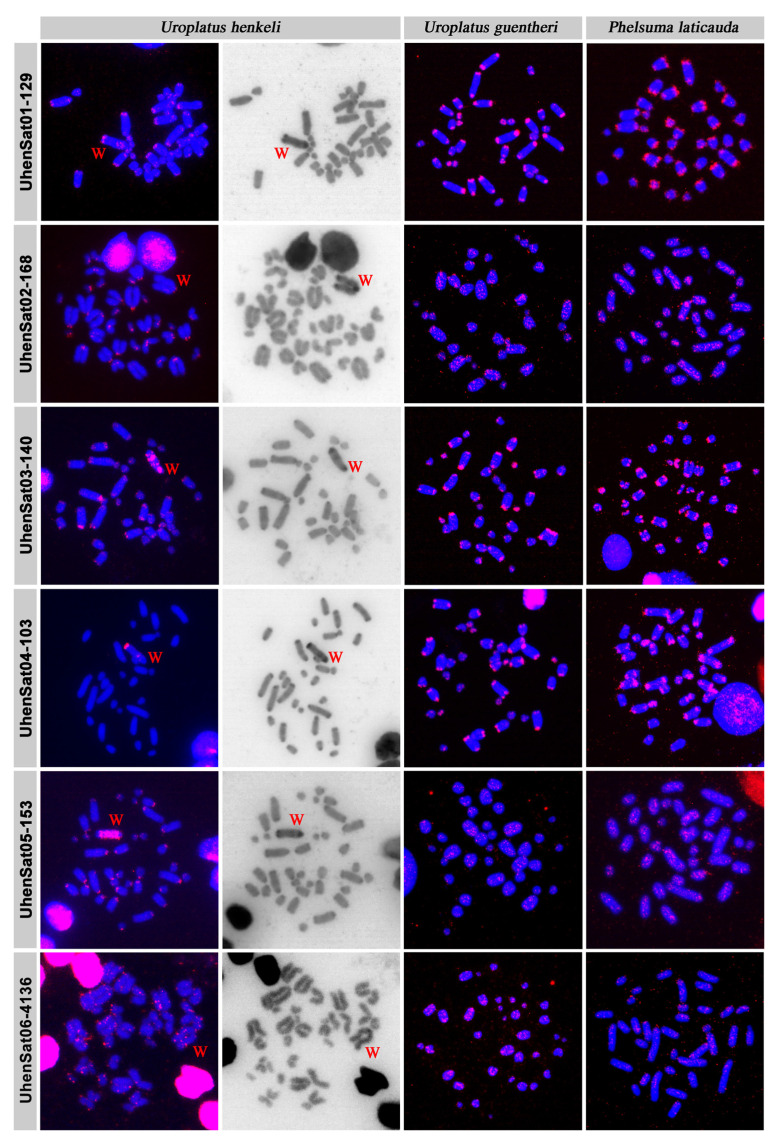
In situ hybridization of satellite DNA motifs UhenSat01-129, UhenSat02-168, UhenSat03-140, UhenSat04-103, UhenSat05-153 and UhenSat06-4136 of *U. henkeli* at metaphases of *U. henkeli*, *U. guentheri* and *P. laticauda*. The W chromosome in *U. henkeli* was identified by C-banding, conducted in chromosome spreads after in situ hybridization.

**Figure 3:**
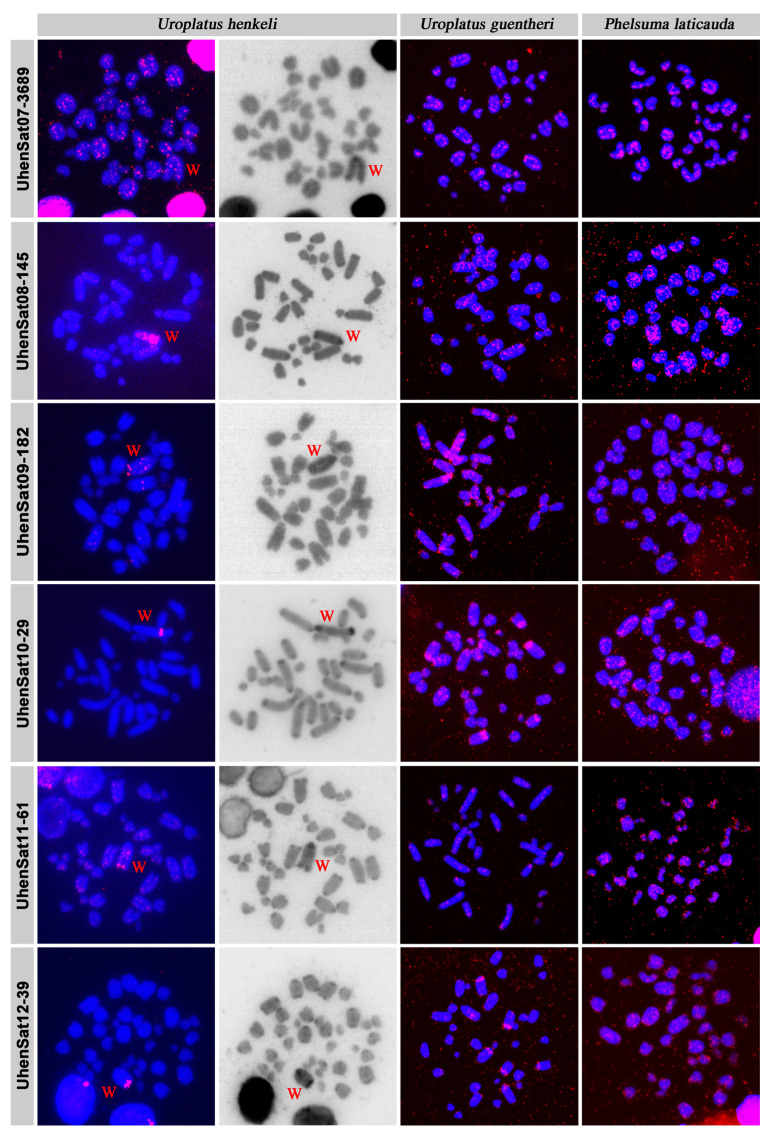
In situ hybridization of satellite DNA motifs UhenSat07-3689, UhenSat08-145, UhenSat09-182, UhenSat10-29, UhenSat11-61 and UhenSat12-39 of *U. henkeli* at metaphases of *U. henkeli*, *U. guentheri* and *P. laticauda*. The W chromosome in *U. henkeli* was identified by C-banding, conducted in chromosome spreads after in situ hybridization.

**Figure 4:**
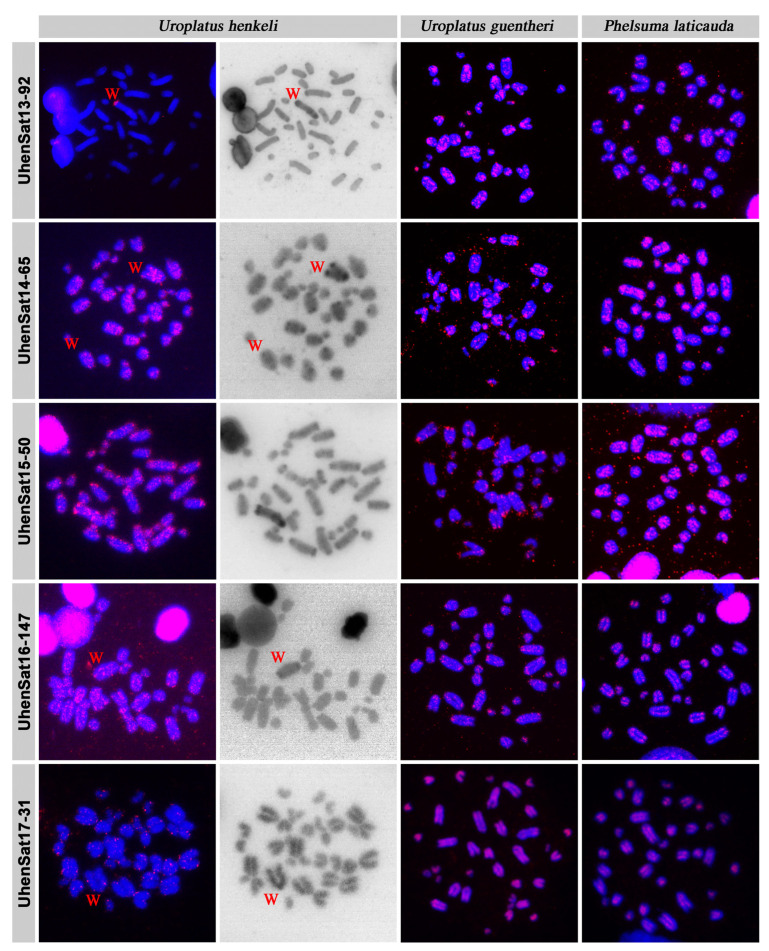
In situ hybridization of satellite DNA motifs UhenSat13-92, UhenSat14-65, UhenSat15-50, UhenSat16-147 and UhenSat17-31 of *U. henkeli* at metaphases of *U. henkeli*, *U. guentheri* and *P. laticauda*. The W chromosome in *U. henkeli* was identified by C-banding, conducted in chromosome spreads after in situ hybridization.

The families of the satellitome of *U. henkeli* show a mean nucleotide divergence value of 11.64% (Table 1), and the nucleotide divergence varied between 4.25% at UhenSat02-168 and 20.82% at UhenSat12-39. This variation demonstrated a different degree of homogenization for each satellite family. The satellite landscape is a good representation of both homogenization and degeneration status of each satDNA. We present the satDNA landscape by plotting abundance versus divergence of satDNA sequences against their consensus sequence for all 17 identified satellites (Figure S2) as well as the individual landscape for each satDNA family (Figure 1). Some satDNA families showed two peaks in the satellite landscape analysis (UhenSat04-103, UhenSat06-4136, UhenSat09-182 and UhenSat15-50), indicating variation or even the presence of possible subfamilies (Figure 1). We designed probes for all 17 identified satDNAs (Table S2), and their topology in the genome was tested by FISH in three species of geckos: *U. henkeli* and *U. guentheri* with ZZ/ZW sex chromosomes and *P. laticauda* with temperature-dependent sex determination (Figure 2, Figure 3 and Figure 4). We verified the homology between sex chromosomes of the two studied *Uroplatus* species as all the tested primer pairs for Z-specific genes in *U. henkeli* also show a Z-specific pattern in *U. guentheri* with a normalized female-to-male gene copy ratio between 0.39 and 0.65 for all six examined genes (Table S3).

The in situ hybridization in *U. henkeli* verified the presence and revealed the topology of the 17 identified satDNAs, while the C-banding stain allowed for us to identify the satDNAs that accumulate in the W chromosome (Figure 2, Figure 3 and Figure 4). Specifically, six satDNAs show a scattered hybridization signal of variable intensity across all chromosomes including the W (UhenSat06-4136, UhenSat07-3689, UhenSat14-65, UhenSat15-50, UhenSat16-147 and UhenSat17-31) (Figure 2, Figure 3 and Figure 4). Two satDNAs also present a scattered hybridization signal (UhenSat08-145, UhenSat11-61) in most of the chromosomes but are highly accumulated in the W chromosome along the entire chromosome (UhenSat08-145) or in two interstitial and telomeric bands (UhenSat11-61) (Figure 3). Two satDNAs (UhenSat01-129 and UhenSat03-140) are located at both ends, telomeric and centromeric regions, in the majority of the chromosomes, showing accumulation in the W chromosomes in the telomere, centromere and one interstitial band (UhenSat01-129) or as several intense bands (UhenSat03-140) (Figure 2). Two satDNAs are located only at the centromeric region of all chromosomes (UhenSat02-168 and UhenSat05-153); on the W chromosome, one is exclusively centromeric (UhenSat02-168), and the other is extensively accumulated along the entire chromosome (UhenSat05-153) (Figure 2). Five satDNAs are almost exclusive of the W chromosome, located in the telomeric region and one interstitial band (UhenSat04-103 and UhenSat09-182), only on the telomeric region (UhenSat12-39 and UhenSat13-92) or in one prominent interstitial band (UhenSat13-92) (Figure 2, Figure 3 and Figure 4). Hence, all the satDNAs described here were located on the W chromosome; notable accumulation of some satDNA was identified on the heterochromatic regions, especially in regions that are stained stronger with C-banding, such as the centromeric and telomeric regions and an interstitial band near the centromere, but some of them showed extensive accumulation across almost the whole chromosome (UhenSat03-140, UhenSat05-153 and UhenSat08-145) (Figure 2 and Figure 3).

**Figure 5:**
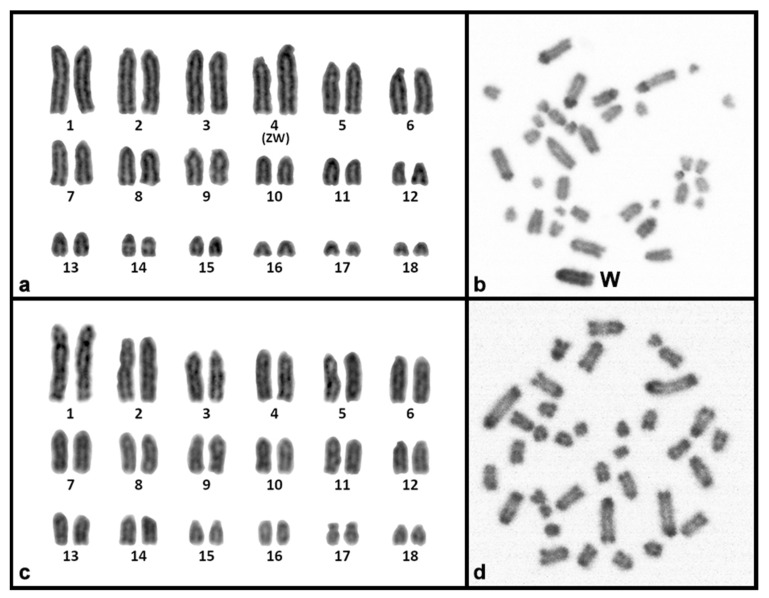
Karyogram and C-banded metaphase from the Günther’s flat-tail gecko *U. guentheri* (**a**,**b**) and gold dust day gecko *P. laticauda* (**c**,**d**). When visible, the W chromosome is indicated.

The karyotype of *U. guentheri* consists of 2n = 36 acrocentric chromosomes. The Z chromosome appears to be among the pairs 4 to 6, as an uneven number of similar-sized chromosomes were detected between sexes. Therefore, we assign, as sex chromosomes in the karyograms, the fourth chromosome pair (Figure 5). C-banding stain revealed the W chromosome, which is slightly larger than the Z. In addition, C-banding strongly stained the centromeric regions of all chromosomes (Figure 5). The in situ hybridization revealed that 14 satDNAs show a scattered hybridization signal across all chromosomes (UhenSat02-168, UhenSat05-153, UhenSat06-4136, UhenSat07-3689, UhenSat08-145, UhenSat09-182, UhenSat10-29, UhenSat11-61, UhenSat12-39, UhenSat13-92, UhenSat14-65, UhenSat15-50, UhenSat16-147 and UhenSat17-31), although some of them are slightly more prominent in the telomeric and centromeric regions (Figure 2, Figure 3 and Figure 4). Three satDNAs (UhenSat01-129, UhenSat03-140 and UhenSat04-103) are clearly located at both centromeric and telomeric regions in the majority of the chromosomes (Figure 2). The localization on the W chromosome of satDNAs was not determined as C-banding was not performed after FISH in *U. guentheri*; therefore, the W chromosome cannot be accurately identified.

The karyotype of *P. laticauda* consists of 2n = 36 acrocentric chromosomes. C-banding stain revealed prominent heterochromatic blocks at the centromeric and telomeric regions of all chromosomes (Figure 5). The in situ hybridization experiments in *P. laticauda* revealed that three satDNAs (UhenSat01-129, UhenSat03-140 and UhenSat04-103) are located at both the telomeric and centromeric regions in the majority of the chromosomes (Figure 2). In addition, the remaining 14 satDNAs show a scattered hybridization signal across all chromosomes (Figure 2, Figure 3 and Figure 4).

## 4. Discussion

We analyzed the satellitome of *U. henkeli*, and we identified 17 families of satDNAs with variability in abundance, monomer length, diversity, A–T content and chromosome distribution. In relation to chromosomal localization, it is remarkable that, in addition to heterochromatin localization, several satDNA families distribute abundantly in the euchromatic regions of chromosomes. This is in accordance with recent studies that demonstrated that satDNA families can also be dispersed along the euchromatin regions of chromosomes [5,6,82]. A recent review in several animal and plant species demonstrated that satellitomes varied highly in the percentage of the genome, number of satDNA families, distribution across the genome and array length [79].

Satellite DNA motifs may exist in long tandem arrays in every part of the genome, which could be located on heterochromatic and euchromatic regions [8,79]; this is in accordance with our findings in the three examined species of geckos: *U. henkeli*, *U. guentheri* and *P. laticauda*. In *U. henkeli*, excluding the W chromosome, eight of the satDNAs are distributed along all the chromosomes, four are on either the centromeric regions or centromeric and telomeric regions of chromosomes, and five show a scattered signal on multiple chromosomes. Regarding the W chromosome, all the satDNA families are present in a variable pattern from a single band to highly accumulated in the entire chromosome (Figure 2, Figure 3 and Figure 4). However, variation was observed in the hybridization pattern for particular satDNA families between *U. henkeli*, *U. guentheri* and *P. laticauda*.

We observed that three satDNAs (UhenSat01-129, UhenSat03-140 and UhenSat04-103; Figure 2) tend to accumulate in both the centromeric and the telomeric regions in *U. guentheri* and *P. laticauda* with two satDNAs (UhenSat01-129 and UhenSat03-140; Figure 2) being common among all three species and abundant in both the telomeric and centromeric regions of all chromosomes. Both centromeres and telomeres are essential structures of the genome. Centromeres are the binding points for the spindle microtubules that guide the chromosome segregation during mitosis and meiosis [83,84], while telomeres stabilize the ends of linear chromosomes and protect them from gradual degradation due to replication [85]. Despite that the telomeric regions show long arrays of the TTAGGG motif in vertebrates, subtelomeric and centromeric regions show an extensive variability in sequence content [86,87]. Centromeric sequences are notorious for their (i) fast evolution rates; (ii) variability in sequence composition, even among chromosomes of the same individual; (iii) diversity in size, shape and organization and (iv) involvement on chromosomal rearrangements, such as Robertsonian fusions, fissions and whole-arm translocations (WARTs), which can shape the karyotype morphology [7].

Geckos have a distinct karyotype morphology among squamate reptiles (except lacertids); they do not possess a “bimodal” karyotype with a mixture of macrochromosomes and microchromosomes, and their karyotypes are dominated completely or mostly by acrocentric chromosomes [88] (Figure 1). We cannot safely reconstruct if the “all-acrocentric” or the “bimodal” karyotype was ancestral for squamates due to the unresolved phylogenetic relationships and conflicting topologies of the geckos in comparison to dibamids and the rest of the squamates [89,90,91,92,93,94,95,96]. Nevertheless, the ancestral karyotype of geckos appears to be 2n = 38 with all-acrocentric chromosomes gradually decreasing in size [97,98,99,100], a trait visible in the majority of the extant species despite their old radiation with a basal split dated between 60 and 170 million years [101]. We speculate that this notable evolutionary stasis might be explained by the presence of satellite motifs at the centromeres that are strong meiotic drivers. Female meiotic drive is the phenomenon of the preferential segregation of certain chromosomes towards the egg than the polar body at meiotic division during female gametogenesis, often causing a significant distortion from the expected ratios of Mendelian inheritance [102]. Such biased segregation can be caused by differences in centromeric sequence content between the two homologous chromosomes. In fact, certain types of satellite motifs can be strong meiotic drivers because they show higher affinity to CENP-A/CENP-B proteins, leading to “stronger” centromeres that generate more attachment sites for microtubules and increase their probability to migrate towards the egg pole [103,104,105]. We assume that a karyotype with “strong” centromeres can remain relatively stable in the long-term because many common chromosome rearrangements altering karyotype morphology (e.g., Robertsonian translocations) tend to severely affect the structure of centromeres [21,106,107] and will not propagate to the egg due to meiotic drive. Therefore, the long-term stability of all-acrocentric karyotypes in geckos might be explained from the presence of centromeres, which are strong meiotic drivers. This hypothesis is further supported by the fact that all three studied species share certain common satellite motifs in all chromosomes of their karyotype. Our hypothesis could be tested in the future by exploring the topology of these satellite motifs among phylogenetically informative species of geckos, covering lineages with all-acrocentric karyotypes but also with bi-armed chromosomes.

Our comparative study revealed that the distribution of several satellite motifs upon the karyotype may differ significantly among species. In fact, 12 satellite families in *U. henkeli* show extensive accumulations in the heterochromatic regions of centromeres, telomeres and the W chromosome, but fewer satellite families show a clear accumulation in *U. guentheri*, and only three show a clear accumulation in *P. laticauda*, a species without sex chromosomes (Figure 2, Figure 3 and Figure 4). Degenerated W/Y chromosomes of vertebrates are often enriched in satellite motifs [49,56,65,66]. Both *U. henkeli* and *U. guentheri* share a homologous ZZ/ZW sex-determination system and similar morphology of the W chromosome in C-banded metaphases, and therefore, we would expect similar sequence content. On the contrary, we observed that different types of satellite motifs are accumulated in each species. It seems that, after roughly 44 million years of divergence since the emergence of this sex-determination system [101], the W chromosomes have followed different paths of independent degeneration and amplification of satellite motifs between species. A similar pattern of interspecies variability of degenerated Y/Ws has been observed in other reptile species with homologous sex-determination systems. For example, all lacertids have homologous ZZ/ZW sex determination with very similar Z-specific gene content [62,66] but variable W according to morphology, heterochromatin distribution and content of microsatellites [66]. Similarly, the Z chromosomes share homologous gene content among all tested caenophidian snakes, but extensive variability was described in the morphology and microsatellite content in the W chromosomes [56].

## Figures and Tables

**Table 1:** Description of satDNA families found in *U. henkeli*: genome proportion (%), the repeat unit length, A + T percentage (%), previous repetitive described.

Satellite Name	Genome Proportion	Repeat Unit Length (bp)	A + T Percentage	Kimura Divergence
UhenSat01-129	0.474	129	43.4	13.59
UhenSat02-168	0.426	168	66.7	4.25
UhenSat03-140	0.287	140	45.1	19.39
UhenSat04-103	0.285	103	47.6	18.53
UhenSat05-153	0.24	153	46.1	9.56
UhenSat06-4136	0.237	4136	43.7	16.36
UhenSat07-3689	0.203	3689	63.1	6.26
UhenSat08-145	0.151	145	56.6	8.92
UhenSat09-182	0.061	182	43.4	13.15
UhenSat10-29	0.055	29	55.2	9.11
UhenSat11-61	0.034	61	70.5	9.80
UhenSat12-39	0.031	39	54.1	20.82
UhenSat13-92	0.024	92	44.6	6.92
UhenSat14-65	0.014	65	48.4	9.30
UhenSat15-50	0.005	50	58	16.13
UhenSat16-147	0.004	147	52.1	6.76
UhenSat17-31	0.002	31	41.9	9.01
Total:	2.534			
Mean:	0.149	550.5	51.8	11.64

## Data Availability

The raw Illumina reads were deposited in the NCBI database under the BioProject PRJNA1027145. All other data are provided directly in the tables, figures and Supplementary Materials of the manuscript.

## Supplementary Materials

The following supporting information can be downloaded at: https://www.mdpi.com/article/10.3390/genes15040429/s1, Figure S1: Chromosome painting with probe from the microdissected W chromosome of *U. henkeli.* The W chromosome is indicated. The probe strongly stains only the W chromosome, which confirms the specificity of the microdissection; Figure S2: Summary of satellitome landscape of *U. henkeli* showing the comparative abundance of the 17 satDNA families as the proportion of the female genome for each satDNA family versus the Kimura substitution divergence (both as percentages); Table S1: Geneious Prime map-to-reference parameters; Table S2: Sequences of identified satellite motifs from *U. henkeli*, the sequences of the designed probes for in situ hybridization and the results of their analyses; Table S3: Results of the qPCR test of homology of sex chromosomes between *U. henkeli* and *U. guentheri*.
