# Supplementary figures and images for: Cytogenetic Analysis of Satellitome of Madagascar Leaf-Tailed Geckos
Source: Genes (Basel). 2024 Mar 28;15(4):429. doi: 10.3390/genes15040429 (PMC11049218; doi:10.3390/genes15040429)

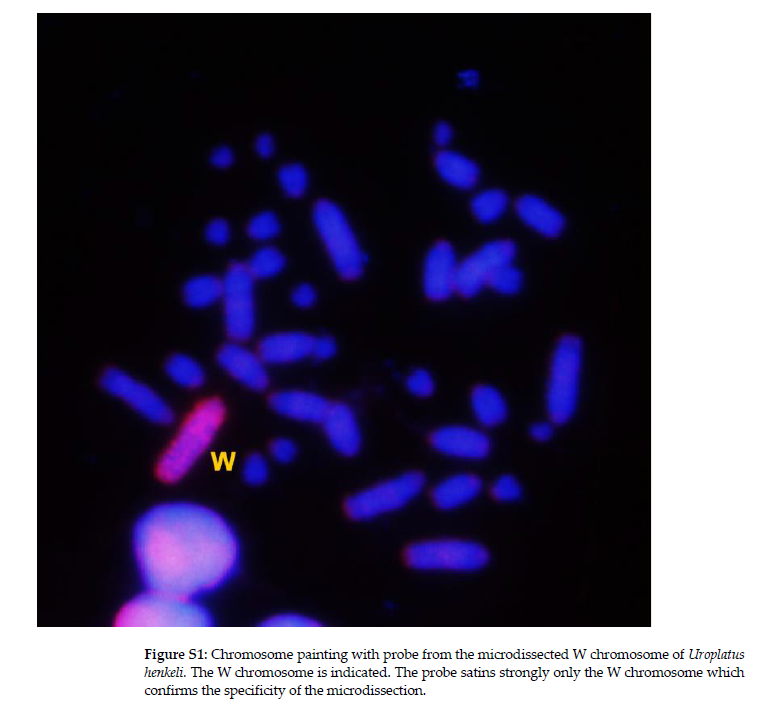

Supplement: Supplementary file 1 [file genes-15-00429-s001.zip › figure S1.png]

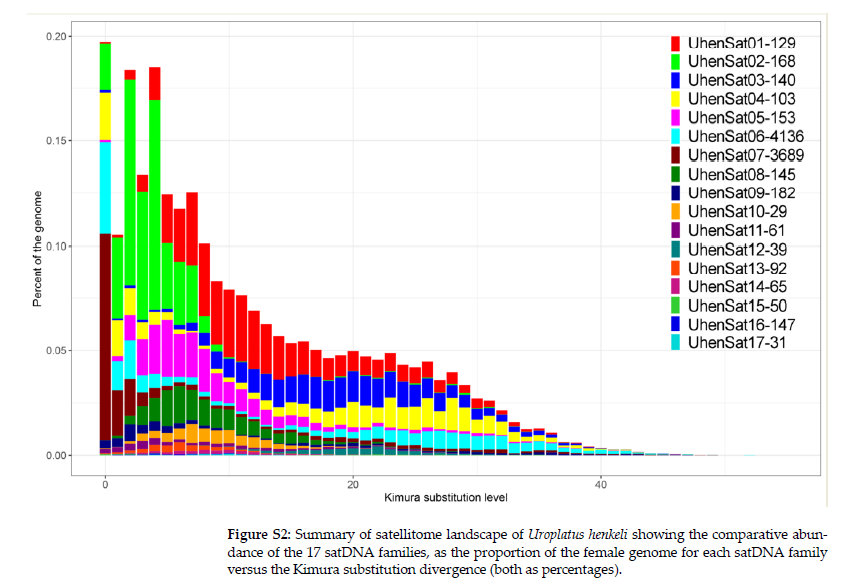

Supplement: Supplementary file 1 [file genes-15-00429-s001.zip › figure S2.png]
